# Melatonin inhibits apoptotic cell death induced by *Vibrio vulnificus* VvhA via melatonin receptor 2 coupling with NCF-1

**DOI:** 10.1038/s41419-017-0083-7

**Published:** 2018-01-19

**Authors:** Sei-Jung Lee, Hyun Jik Lee, Young Hyun Jung, Jun Sung Kim, Sang Ho Choi, Ho Jae Han

**Affiliations:** 1 Department of Pharmaceutical Engineering, Daegu Haany University, Gyeongsan, 38610 South Korea; 2 Department of Veterinary Physiology, College of Veterinary Medicine, Research Institute for Veterinary Science, and BK21 PLUS Program for Creative Veterinary Science Research, Seoul National University, Seoul, 08826 South Korea; 3 National Research Laboratory of Molecular Microbiology and Toxicology, Department of Agricultural Biotechnology, and Center for Food Safety and Toxicology, Seoul National University, Seoul, 08826 South Korea

## Abstract

Melatonin, an endogenous hormone molecule, has a variety of biological functions, but a functional role of melatonin in the infection of Gram-negative bacterium *Vibrio vulnificus* has yet to be described. In this study, we investigated the molecular mechanism of melatonin in the apoptosis of human intestinal epithelial (HCT116) cells induced by the hemolysin (VvhA) produced by *V. vulnificus*. Melatonin (1 μM) significantly inhibited apoptosis induced by the recombinant protein (r) VvhA, which had been inhibited by the knockdown of MT_2_. The rVvhA recruited caveolin-1, NCF-1, and Rac1 into lipid rafts to facilitate the production of ROS responsible for the phosphorylation of PKC and JNK. Interestingly, melatonin recruited NCF-1 into non-lipid rafts to prevent ROS production via MT_2_ coupling with Gαq. Melatonin inhibited the JNK-mediated phosphorylation of c-Jun responsible for Bax expression, the release of mitochondrial cytochrome c, and caspase-3/-9 activation during its promotion of rVvhA-induced apoptotic cell death. In addition, melatonin inhibited JNK-mediated phosphorylation of Bcl-2 responsible for the release of Beclin-1 and Atg5 expression during its promotion of rVvhA-induced autophagic cell death. These results demonstrate that melatonin signaling via MT_2_ triggers recruitment of NCF-1 into non-lipid rafts to block ROS production and JNK-mediated apoptotic and autophagic cell deaths induced by rVvhA in intestinal epithelial cells.

## Introduction

*Vibrio vulnificus* is a halophilic Gram-negative bacterium frequently found in warm coastal waters. The extent of *V. vulnificus-*related disease can range from gastroenteritis to primary sepsis (vibriosis) in high-risk individuals^1^. Infection of *V. vulnificus* is cytotoxic to host cells, and its virulence is mediated by secreted cytotoxins and enzymes, such as VvhA, MARTX, and Vvp^2–4^. Although virulence effects of VvhA were controversial in an in vivo of cells infected with a VvhA mutant, VvhA, a pore-forming toxin, is known for its cytolytic activity against erythrocytes and host cells^5^. It has also been suggested that the balance of intracellular ROS in host cells during microbial infection regulates not only the elimination of phagocytosed microbes but also the signaling cascades related to inflammation and immune responses^6,7^. Recent studies have suggested that rVvhA increases ROS production through the lipid raft-dependent c-Src/NOX signaling pathway^8^. Specifically, *V. vulnificus* infection increases ROS-dependent p38/NOX signaling and host cell death^9^. Therefore, as host cell death induced by VvhA is provoked by formation of ROS, investigation of approaches to maintain an appropriate ROS level and prevent excessive ROS accumulation is required.

ROS derived from NOX2 complex has influential roles in regulating inflammation, host defense, and inducing cell apoptosis against bacterial infection^10^. The NOX2 activation mechanism has been described as having three different activation states: resting, primed, and activated, which are controlled by the change of the subcellular localization of regulatory subunits. NCF-1 is a 47 kDa cytosolic subunit of NADPH which is required for activation of the NOX2 to produce the superoxide anion^11^. In response to pathogens, p47^*phox*^/NCF-1 is translocated to the lipid rafts of plasma membrane, which interacts with the catalytic subunits of the NOX^12–14^. Moreover, our previous reports suggest that regulation of the NOX2 complex by *V. vulnificus* has a potential role in ROS regulation and cell death^13^. Although the roles of ROS in microbial pathogenesis and host defense have not been fully described, further investigation into the identification of detailed regulatory mechanism of NOX-induced ROS production in host cells may provide a potential therapeutic strategy for protecting against cytotoxic damage caused by the *V. vulnificus* infection^15^.

Melatonin (N-acetyl-5-methoxytryptamine) is an endogenous hormone produced in the pineal gland and non-neural tissue that has a capacity to control cell physiology and function, and its physiological actions are mediated by membrane-bound melatonin receptors MT1 and MT_2_^16–18^. Antioxidative action of melatonin is achieved through a variety of inducements of antioxidant enzymes, inhibition of pro-oxidant enzymes, maintenance of mitochondrial ROS homeostasis, and direct scavenging of free radicals^17,19^. Previous researchers have reported on the protective activity of melatonin against infection by several bacteria, such as *Chlamydia pneumoniae*, *Mycobacterium tuberculosis*, and *Pseudomonas aeruginosa*, but the effect of melatonin on *V. vulnificus* infection has not been reported^20,21^. Although antibacterial effects of melatonin have been assessed in different types of bacteria, the specific mechanism involved and the virulence factors with an influential effect in host cells during intestinal infection remain incompletely described. Thus, in this study, we investigated the role of melatonin in controlling NOX2-produced ROS by VvhA challenge and the protective effect of melatonin in VvhA-induced intestinal host cell apoptosis.

## Materials and Methods

### Materials

Fetal bovine serum (FBS) was purchased from Hyclone (Logan, UT, USA). The following antibodies were purchased: Rac1 antibody (BD Biosciences, Franklin Lakes, NJ, USA); c-Jun N-terminal kinase (JNK), p-JNK, p-p38, p38, p-ERK, ERK, p-PKC, PKC, p-c-Src, c-Src, p-NF-κBp65, NF-κBp65, p-c-Jun, c-Jun, Bax, p-Bcl-2, Bcl-2, caveolin-1, cleaved caspase-3, caspase-9, and β-actin antibodies (Santa Cruz Biotechnology, Paso Robles, CA, USA); Bax (6A7) monoclonal antibody (Thermo Fisher Scientific, Rockford, IL, USA); LC-3, NCF-1 and Beclin-1 antibodies (Novus Biologicals, Littleton, CO, USA). VvhA-specific antibody was acquired from Professor Sang Ho Choi (Seoul National University, Seoul, Korea). Horseradish peroxidase (HRP)-conjugated goat anti-rabbit and goat anti-mouse IgG antibodies (Jackson Immunoresearch, West Grove, PA, USA). SP600125 was purchased from Sigma-Aldrich (St. Louis, MO, USA). All other reagents were of the highest purity commercially available and were used as received.

### Cells

HCT116 colon cancer epithelial cells were purchased from the American Type Culture Collection (ATCC, Manassas, VA, USA) and cultured at 37 °C in 5% CO_2_ in McCoy’s 5 A medium containing 10% FBS and antibiotics. INT407 cells were kindly provided by Professor Sang Ho Choi and were grown in α-Minimum Essential Medium supplemented with 10% FBS and antibiotics.

### Purification of the recombinant protein (r) VvhA

To identify the functional role of VvhA in HCT116 cells, we prepared a recombinant protein of VvhA (rVvhA). The oligonucleotides were designed by using the *V. vulnificus* MO6-24/O genomic sequence (GenBank™ accession number CP002469 and CP002470, www.ncbi.nlm.nih.gov)^22^. Briefly, the open reading frame of VvhBA was amplified by performing PCR with a pair of primers for VvhA (Supplemental Table 1) and cloned into a His6 tag expression vector, pET29a( + ) (Novagen, Madison, WI, USA) to result in pKS1201 (Supplemental Table 2). *Escherichia coli* BL21 (DE3) harboring pKS1201 was grown in LB-ampicillin medium at 37 °C until the cultures reached an A_600_ between 0.5 and 0.6. The temperature was then lowered to 30 °C, and protein expression was induced by treatment with 1 mM isopropyl-β-D-thiogalactopyranoside (IPTG) for 6 h. The cells were harvested by centrifugation at 5,000 × *g* for 20 min at 4 °C. The cell pellets were resuspended in buffer A (20 mM Tris-Cl, pH 8.0, and 500 mM NaCl), and the cell suspensions were ultrasonicated. The crude cell extracts were centrifuged at 16,000 × *g* for 30 min at 4 °C, and the supernatant was filtered by using a 0.2 μm Whatman Puradisc syringe filter (Whatman International, Maidstone, Kent, UK) to isolate the soluble fraction. Cell lysate containing His6-tagged VvhBA protein was mixed with 1 mL of nickel-nitrilotriacetic acid agarose (Qiagen, Valencia, CA, USA) for 1 h at 4 °C, and the mixture was loaded on Bio-Spin® chromatography columns (Bio-Rad Laboratories, Hercules, CA, USA). The resin was washed with buffer A, and the bound VvhBA protein was eluted with buffer A containing 300 mM imidazole. After purification, the homogeneity of VvhBA was assessed by 12% sodium dodecyl sulfate-polyacrylamide gel electrophoresis (SDS-PAGE) and Coomassie blue staining. Purified proteins were dialyzed against 20 mM Tris-Cl, pH 8.0, concentrated to 0.3 mg/mL by using Slide-A-Lyzer dialysis cassettes (Thermo Scientific, Hudson, NH, USA) and stored at −80 °C until use.

### Cell viability measurement

Cell viability assay was performed by using EZ-CYTOX cell viability commercial kit (Dail Labservice, Seoul, Korea) according to the manufacturer’s instructions. Cells were cultured on 96-well culture plates. After incubation with melatonin or rVvhA, EZ-CYTOX master mix was added to each well. After incubation for 1 h, cell viability was analyzed by measurement absorbance at 450 nm.

### Flow cytometry

To quantify the number of rVvhA-induced cell death, cells were synchronized in the G_0_/G_1_ phase by culture in serum-free medium for 24 h before incubation with melatonin and rVvhA and the samples were prepared as described in previous report^23^. rVvhA-induced cell death was detected with an Annexin V and PI staining kit (BD Biosciences, Franklin Lakes, NJ, USA) according to the manufacturer’s instructions. Also, to evaluate the level of mitochondrial ROS in the cells treated with rVvhA and melatonin, MitoSOX, a mitochondrial superoxide indicator, was used to detect the mitochondrial ROS level and analyzed by flow cytometry. Flow cytometry was performed by Cell Lab Quanta SC (Beckman Coulter, Brea, CA, USA) and analyzed by using flowing software 2 (developed by Perttu Terho, Turku, Finland).

### RNA isolation and reverse transcription-polymerase chain reaction (RT-PCR)

Total RNA was extracted by using the RNeasy Plus Mini Kit (Qiagen). Reverse transcription (RT) was carried out with 3 μg of RNA by using a Maxime RT premix kit (iNtRON Biotechnology, Sungnam, Korea). β-actin (*ACTB*) gene was used as an endogenous control. The cDNA (5 µL) for melatonin receptors was amplified as described previously^24^. The primer sequences for *MT*_*1*_, *MT*_*2*_ and *ACTB* were described in Supplemental Table 3.

### Quantitative real-time PCR

The relative mRNA expression level of the *ATG5* was analyzed using a Rotor-Gene 6000 device (Corbett Research, Cambridge, UK) with the QuantiMix SYBR Kit (PhileKorea Technology, Daejeon, Korea) according to the manufacturers’ instructions. The primer sequences for *ATG5* were described in Supplemental Table 3. *ACTB* was used as an endogenous control.

### Small interfering RNA transfection

Cells were grown until 75% coverage of the surface of the plate and then transfected for 24 h with ON-TARGETplus siRNAs mixed with siRNAs specific for MT_2_, NCF-1, Gαi, Gαq, Gα12, PKCα, JNK, Atg5, non-targeting (GE Dharmacon, Lafayette, CO, USA) or Bax (Bioneer, Daejeon, Korea with HiPerFect Transfection Reagent (Qiagen) according to the manufacturer’s instructions. The siRNA efficacy for MT_2_, NCF1, Gαi, Gαq, Gα12, PKCα, JNK, Atg5 and Bax determined by western blot was 71, 69, 76, 75, 69, 71, 61, 68, and 60%, respectively (Supplemental Fig. 1).

### Detergent-free purification of caveolin-rich membrane fraction

Cells were washed twice with ice-cold PBS, scraped into 2 mL of 500 mM sodium carbonate (pH 11.0), transferred to a plastic tube, and homogenized with a Sonicator 250 apparatus (Branson Ultrasonic, Danbury, CT, USA) using three 20 sec bursts. The homogenate was separated by sucrose gradient ultracentrifugation and the preparation of sucrose gradient buffer is described in our previous report^23^. A 5–35% discontinuous sucrose gradient centrifuged at 40,000 × *g* for 20 h in a Beckman SW41 Rotor (Beckman Coulter, Fullerton, CA, USA). Eight fractions were collected and analyzed by 12% SDS-PAGE.

### Western blot analysis

Cells were harvested, washed twice with PBS, and lysed with buffer {20 mM Tris (pH 7.5), 1 mM EDTA, 1 mM EGTA, 1% Triton X-100, 1 mg/mL aprotinin, and 1 mM phenylmethylsulfonyl fluoride (PMSF)}. Protein concentrations were determined by BCA Protein Assay kits (Pierce, Rockford, IL, USA). Sample proteins were resolved by SDS-PAGE and transferred to PVDF membranes. The membranes were incubated with the primary antibody at 4 °C overnight. The membrane was then washed and incubated with a horseradish peroxidase-conjugated secondary antibody for 2 h. The specific bands were visualized by the ChemiDoc XRS + System (Bio-Rad, Richmond, CA, USA).

### Immunoprecipitation

Interactions of either NCF-1 with MT_2_, Rac1, and caveolin-1, or Bcl-2 with Beclin1, NOX2 with VvhA were analyzed by undertaking immunoprecipitation and western blotting. Cell lysates (400 μg) were mixed with 10 μg of each antibody and the antigen/antibody complex was pulled down with Protein A/G PLUS-agarose immunoprecipitation reagent (Pierce, Rockford, IL, USA) as explained previously^23^. Beads were washed four times, and the bound proteins were released from the beads by boiling in SDS–PAGE sample buffer for 5 min. Western blotting was performed as described above.

### Intracellular and mitochondrial reactive oxygen species detection

The CM-H_2_DCFDA and MitoSOX (Thermo Fisher Scientific, Waltham, MA, USA) were used to detect intracellular ROS and mitochondrial ROS, respectively. Intracellular ROS and mitochondrial reactive oxygen species (ROS) levels were detected by a luminometer (Victor3; Perkin-Elmer, MA, USA) according to the manufacturer’s instruction.

### Cytosol and mitochondria fractionation

Isolation of mitochondria from cultured HCT116 cells was performed by using a mitochondria isolation kit (Thermo Fisher Scientific, Rockford, IL, USA) according to the manufacturer’s instructions. For lysis of the isolated mitochondria, 100 μL of 2 % CHAPS in TBS {25 mM Tris, 0.15 M NaCl (pH 7.2)} were added to the mitochondrial pellet and vortexed for 1 min. And then they were centrifuged at 12,000 × *g* for 2 min. The fractions were then subjected to western blotting as described above.

### Immunofluorescence analysis

The autolysosomes and lipid raft were immunostained with LysoTracker Red DND-99 (LysoTracker, Invitrogen Co., Carlsbad, CA, USA) and cholera toxin B subunit (CTB, Sigma-Aldrich), respectively. Immunocytochemical staining was performed as described in our previous report and counterstained with 300 nM DAPI in PBS for 5 min^8^. The immunostained cells were visualized with an Olympus FluoView 300 confocal microscope with 400 × objective. The co-localization of protein with LysoTracekr or CTB was analyzed by using Metamorph software.

### Statistical analysis

Results are expressed as means ± S.E. Experimental results were analyzed by the analysis of variance, and comparing means of treatment groups with that of control was performed by using Student’s t-test. Differences were considered statistically significant at *p* < 0.05.

## Results

### Melatonin prevents apoptosis induced by VvhA

**Fig. 1:**
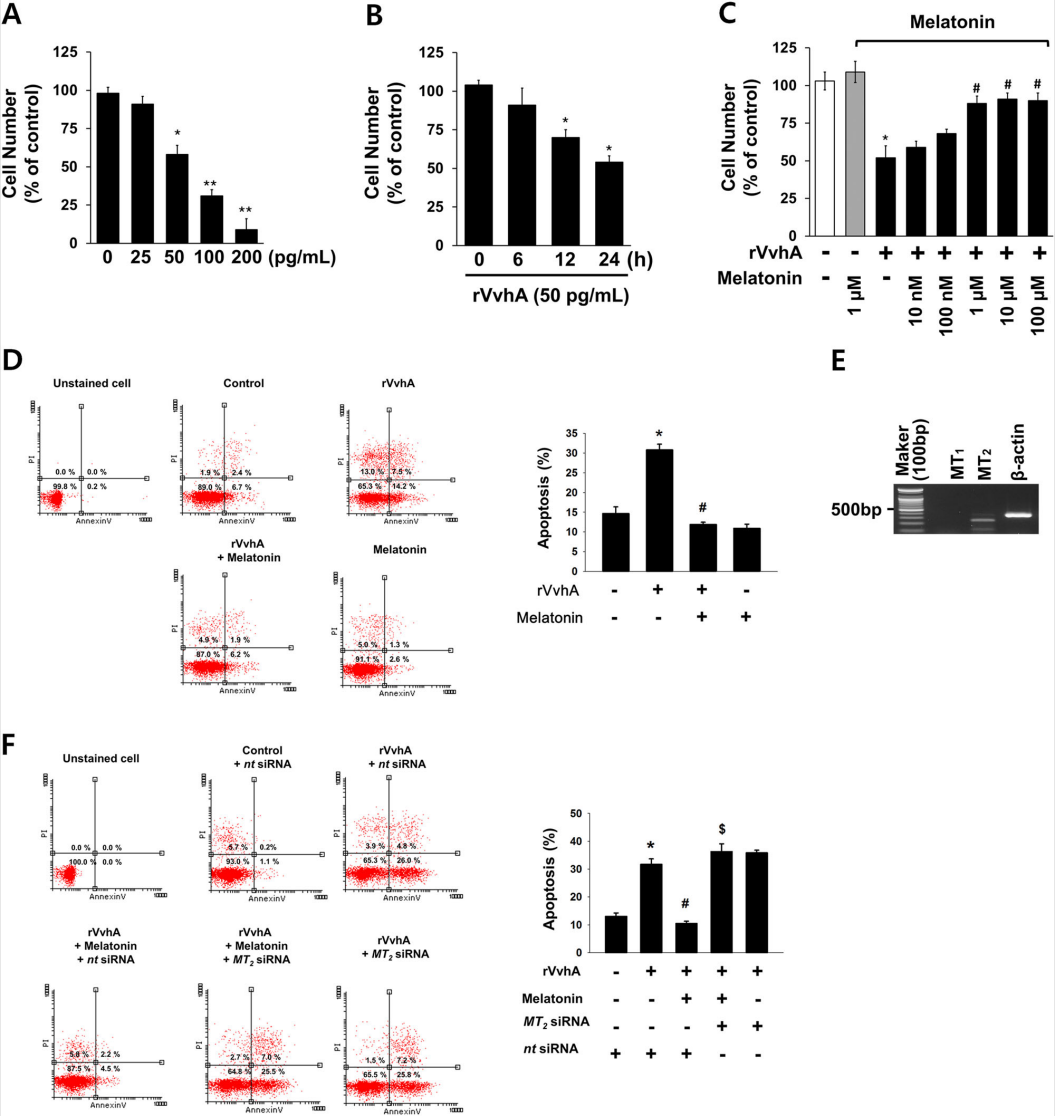
Melatonin prevents apoptosis induced by VvhA via MT_2_ **a** Dose responses of rVvhA for 24 h in cell counting assay are shown. Data represent means ± S.E. *n* = 5. *p < 0.05 vs. 0 pg/mL. **P < 0.01 vs. 0 pg/mL. **b** Time responses of 50 pg/mL of rVvhA in cell counting assay are shown. Error bars represent the means ± S.E. *n* = 5. *p < 0.01 vs. 0 h. **c** Cells were treated with melatonin at various concentrations (10–100 µM) for 30 min prior to rVvhA (50 pg/mL) exposure for 24 h. *p < 0.01 vs. vehicle. ^#^*p* < 0.01 vs. rVvhA alone. **d** Cells were incubated with melatonin (1 µM) for 30 min prior to rVvhA exposure for 24 h. Percentages of total apoptotic cells were measured by using Annexin V/PI staining and flow cytometry. *n* = 4. *p < 0.01 vs. control. ^#^*p* < 0.01 vs. rVvhA alone. **e** Expression of MT_1_ and MT_2_ mRNAs in HCT116 cells. A representative 1% agarose gel following RT–PCR is shown. *n* = 3. **f** Cells transfected with siRNAs for non-targeting (*nt*) control and MT_2_ were incubated with melatonin (1 µM) for 30 min prior to rVvhA exposure for 24 h. Percentages of total apoptotic cells were measured by using Annexin V/PI staining and flow cytometry. *n = *4. *p < 0.01 vs. *nt* siRNA alone. ^#^*p* < 0.01 vs. rVvhA + *nt* siRNA. ^$^*p* < 0.01 vs. rVvhA + melatonin + *nt* siRNA

To confirm that the VvhA produced by *V. vulnificus* used in this study induced cytotoxicity in HCT116 cells, the cells were exposed to various concentrations (0–200 pg/mL) of rVvhA for 24 h. rVvhA treatments from 50 to 200 pg/mL significantly induced cytotoxicity of HCT116 cells (Fig. 1a). An increase in cytotoxicity was observed after 12 h of incubation with 50 pg/mL of rVvhA (Fig. 1b). Pretreatment with melatonin at concentrations of 1 to 100 µM reversed the amount of cell number reduction by rVvhA (Fig. 1c). We further showed that melatonin significantly prevented rVvhA-induced apoptotic cell death (Fig. 1d). And, we confirmed that melatonin pretreatment significantly inhibited VvhA-induced cell death in Caco2 and INT407 cells (Supplemental Fig. 2A) and melatonin pretreatment inhibited rVvhA-induced cleavage of caspase-3 in INT407 cells (Supplemental Fig. 2B). Furthermore, we detected MT_2_ expression, but no MT1 expression (Fig. 1e). The anti-apoptotic effect of melatonin was inhibited by knockdown of MT_2_ expression with *MT*_2_ siRNA in HCT116 cells treated with rVvhA (Fig. 1f). These results indicate that the protective effect of melatonin on *V. vulnificus* infection is related to blockage of apoptosis caused by rVvhA, and that melatonin triggers the MT_2_ receptor-mediated signaling pathway.

### Melatonin induces NCF-1 binding to MT_2_ in non-lipid raft to block ROS production and apoptosis induced by rVvhA

**Fig. 2:**
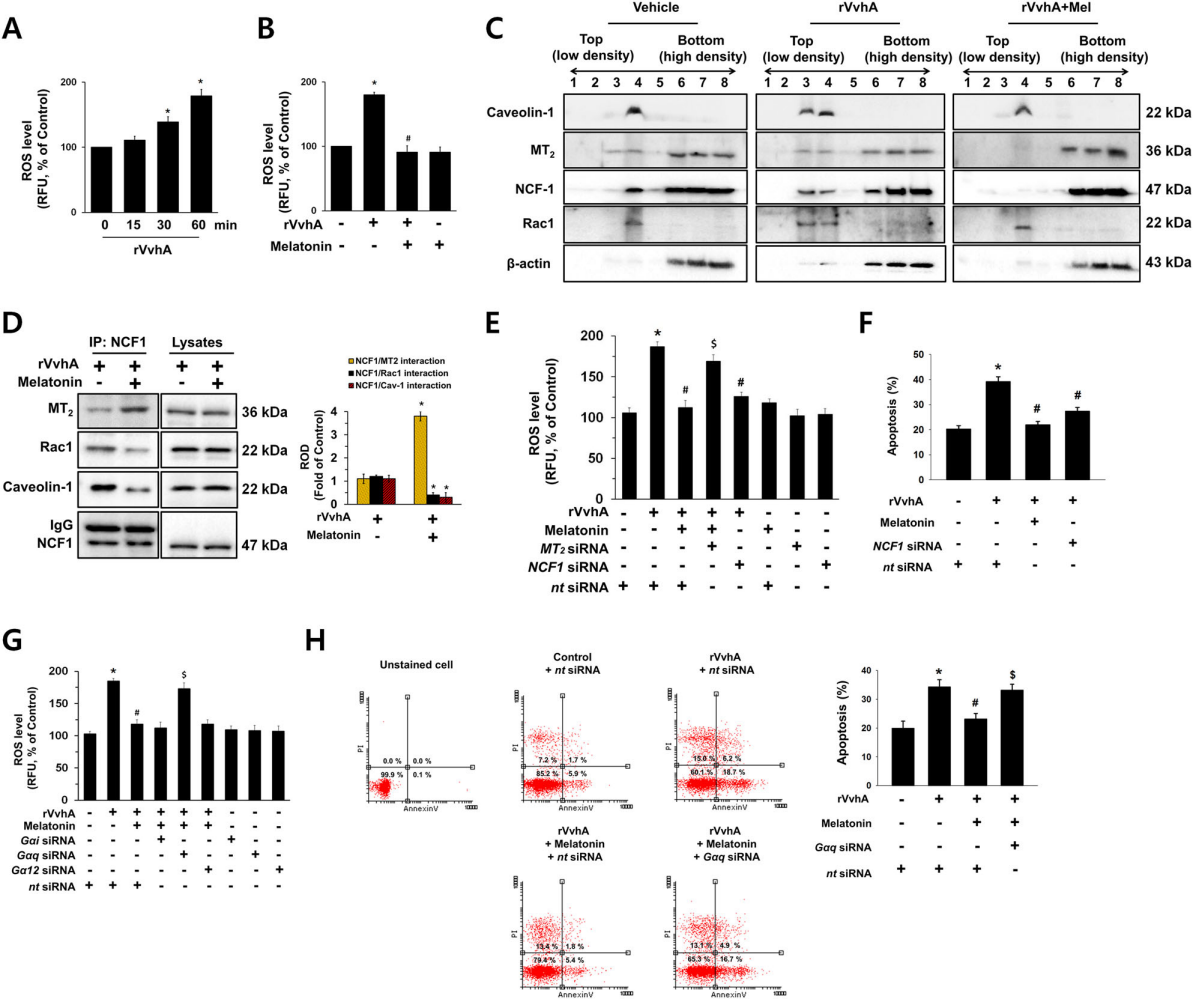
Melatonin induces NCF-1 binding to MT2 in non-lipid raft to block ROS production and apoptosis in HCT116 cells treated with rVvhA **a** The level of ROS production in cells treated with 50 pg/mL of rVvhA for 60 min is shown. Data represent the means ± S.E. *n* = 5. *p < 0.05 vs. 0 min. **b** The level of ROS production in cells treated with melatonin (1 µM) for 30 min prior to rVvhA exposure for 60 min is shown. Data represent the mean ± S.E. *n* = 4. *p < 0.05 vs. control. ^#^*p* < 0.01 vs. rVvhA alone. **c** Caveolin-enriched membrane fractions were prepared by discontinuous sucrose density gradient fractionation from the cell treated with rVvhA for 30 min, and the location of caveolin-1, MT_2_, NCF-1, and Rac1 was determined by western blot. *n* = 3. **d** NCF-1 co-immunoprecipitated with MT_2_, Rac1, and Caveolin-1 (left side). The level of MT_2_, Rac1, Caveolin-1, and NCF-1 in total cell lysates is shown in the right side. *n = *3. *p < 0.01 vs. rVvhA alone. **e** Cells transfected with siRNAs for MT_2_ or NCF-1 were incubated with melatonin (1 µM) for 30 min prior to rVvhA exposure for 60 min. The level of ROS production is shown. Data represent the mean ± S.E. *n* = 4. *p < 0.05 vs. control. ^#^*p* < 0.01 vs. rVvhA alone. ^$^*nt* siRNA < 0.01 vs. rVvhA + melatonin+ *nt* siRNA. **f** Cells transfected with siRNAs for non-targeting (*nt*) control and NCF-1 were incubated with melatonin (1 µM) for 30 min prior to rVvhA exposure for 24 h. Quantitative analysis of the total apoptotic cells by flow cytometer analysis is shown. *n = *3. *p < 0.01 vs. *nt* siRNA. ^*#*^*p* < 0.01 vs. rVvhA + *nt* siRNA. **g** Cells transfected with siRNAs for Gαi, Gαq and Gα12 were incubated with melatonin (1 µM) for 24 h prior to rVvhA exposure for 60 min. The level of ROS production is shown. Data represent the mean ± S.E. *n* = 4. *p < 0.05 vs. *nt* siRNA. ^#^*p* < 0.01 vs. rVvhA + *nt* siRNA. ^$^*p* < 0.01 vs. *nt* siRNA+ rVvhA+ melatonin. **h** Cells transfected with siRNAs for Gαq and *nt* were incubated with melatonin (1 µM) for 24 h prior to rVvhA exposure for 24 h. Quantitative analysis of the percentage of apoptotic cells by flow cytometer analysis is shown. *n = *4. *p < 0.01 vs. *nt* siRNA. ^#^*p* < 0.01 vs. rVvhA + *nt* siRNA. ^$^*p* < 0.01 vs. rVvhA+ melatonin+ *nt* siRNA

We investigated the effect of melatonin on VvhA-induced NOX2-dependent ROS production. Our data shows a significant increase in the ROS level appeared between 0.5 and 1 h after incubation with 50 pg/mL of rVvhA (Fig. 2a), and that increase could be blocked by melatonin treatment (Fig. 2b). Fig. 2c shows that rVvhA markedly induced the recruitment of a lipid raft marker caveolin-1, NCF-1 and Rac1 into fraction 3, but no change in membrane location of MT_2_ was observed (Fig. 2c, middle panel). Melatonin markedly induced the recruitment of MT_2_ and NCF-1 into the non-lipid raft parts (Fig. 2c, right panel). We tried to quantify the results by using co-immunoprecipitation of NCF-1 with MT_2_, Rac1, and caveolin-1 in the presence of rVvhA and melatonin (Fig. 2d). The results revealed that the interaction of NCF-1 with MT_2_ was increased by melatonin treatment. However, it was noted that melatonin attenuates the interaction of NCF-1 with Rac1 and caveolin-1 in rVvhA-treated HCT116 cells. Regarding the VvhA interaction with NOX2 complex, we further performed additional experiments whether VvhA interacts with NOX2 by using polyclonal VvhA antibody to check interaction of VvhA with NOX2 complex. As shown in Supplemental Figs. 3 and 4, we found that VvhA is localized at lipid raft and bind to NOX2 protein. This indicates that VvhA localized at lipid raft to interact with NOX2, where it stimulates the ROS production. In addition, we have shown here that melatonin does not have effect on co-localization between VvhA and CTB, a lipid raft-specific fluorescent dye (Supplemental Fig. 4). NCF-1 silencing suppressed rVvhA-induced ROS production, and silencing of *MT*_*2*_ siRNA significantly abrogated the anti-oxidative effect of melatonin (Fig. 2e). In support of the above results, knockdown of NCF-1 significantly abrogated rVvhA-induced apoptosis (Fig. 2f). Furthermore, we showed that silencing of small GTPase subunit Gαi and Gα12 with siRNAs did not affect the melatonin-dependent anti-oxidative effect, ruling out the role of Gαi and Gα12 (Fig. 2g). However, inhibition of Gαq by *Gαq* siRNA blocked the melatonin-induced anti-oxidative effect. Consistent with those results, knockdown of Gαq significantly abrogated the anti-apoptotic effect of melatonin (Fig. 2h). Taken together, these findings suggest that non-lipid raft-mediated clustering of NCF-1 with MT_2_ induced by melatonin is critical for the inhibition of the NOX2-dependent ROS production and apoptosis triggered by rVvhA treatment.

### Melatonin inhibits PKC/JNK pathway activated by rVvhA

**Fig. 3:**
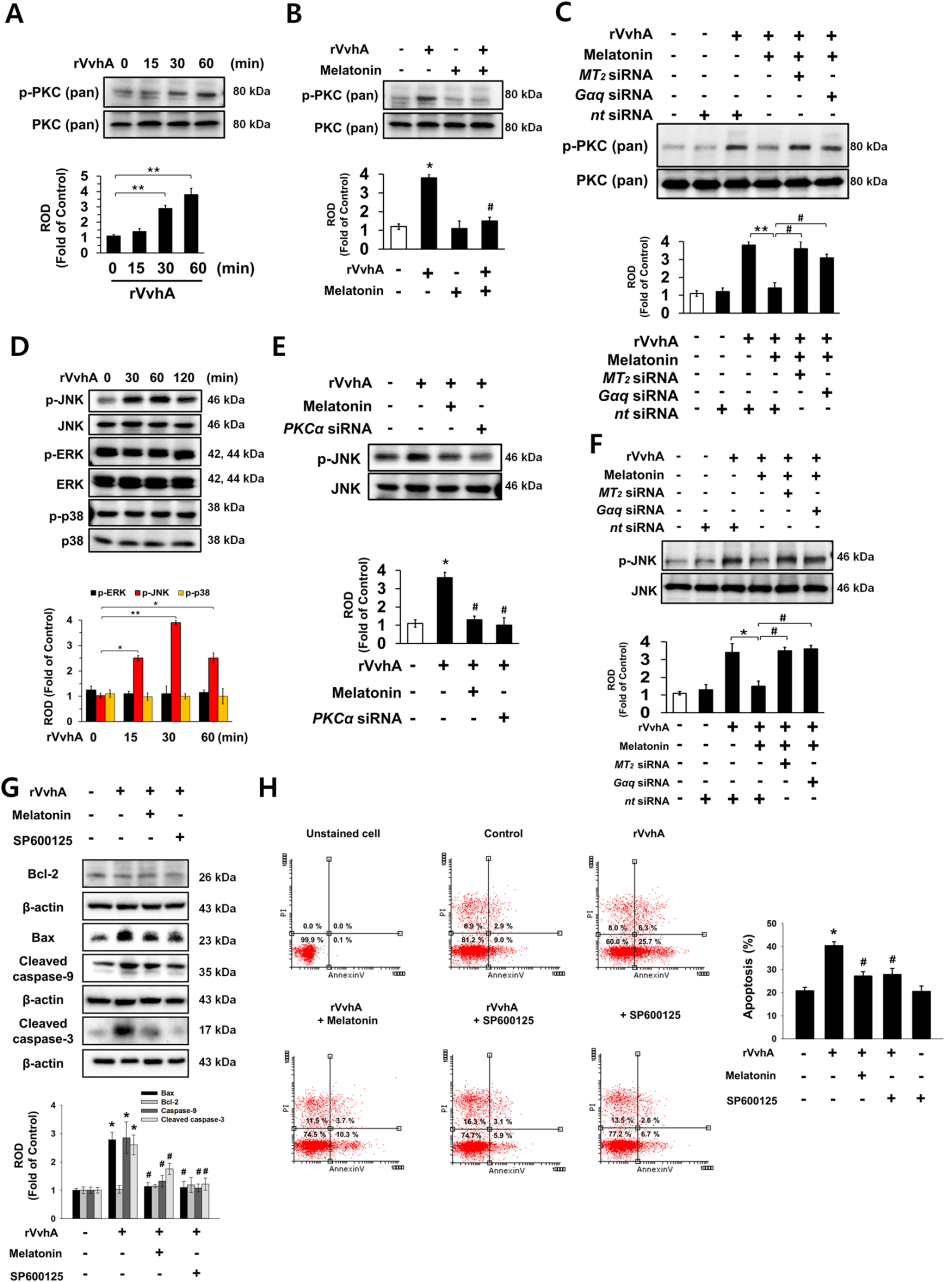
Regulatory effect of melatonin on phosphorylation of PKC and JNK **a** Phosphorylation of pan-PKC is shown *n* = 3. **p < 0.01 vs. control. **b** Phosphorylation of pan-PKC in cells treated with melatonin (1 µM) for 30 min prior to rVvhA exposure for 60 min is shown. *n* = 4. *p < 0.01 vs. control. ^#^*p* < 0.05 vs. rVvhA alone. **c** Cells transfected with siRNAs for MT_2_ and Gαq were incubated with melatonin (1 µM) for 30 min prior to rVvhA exposure for 60 min. The level of PKC phosphorylation is shown. Data represent the mean ± S.E. *n* = 4. **p < 0.01 vs. *nt* siRNA. ^#^*p* < 0.01 vs. rVvhA+ melatonin+ *nt* siRNA. **d** The effect of rVvhA on the expression of MAPK was determined by western blot. *n* = 3. *p < 0.05 vs. 0 min. **p < 0.01 vs. 0 min. **e** Cells transfected with siRNA for PKCα were incubated with melatonin (1 µM) for 30 min prior to rVvhA exposure for 60 min. The level of JNK phosphorylation is shown. Data represent the mean ± S.E. *n* = 4. *p < 0.01 vs. control. ^#^*p* < 0.05 vs. rVvhA alone. **f** Cells transfected with siRNAs for MT_2_ and Gαq were incubated with melatonin (1 µM) for 30 min prior to rVvhA exposure for 60 min. The level of JNK phosphorylation is shown. Data represent the mean ± S.E. *n* = 4. *p < 0.01 vs. *nt* siRNA. ^#^*p* < 0.01 vs. rVvhA+ melatonin+ *nt* siRNA. **g**, **h** Cells were pretreated with SP600125 (10 μM) or melatonin (1 μM) for 30 min prior to rVvhA exposure for 24 h. Bcl-2, Bax, caspase-9 and cleaved caspase-3 were detected by western blot. Data represent the mean ± S.E. *n* = 4. *p < 0.05 vs. control. ^#^*p* < 0.01 vs. rVvhA alone. **h** Quantitative analysis of the percentage of apoptotic cells by flow cytometer analysis is shown. *n = *4. *p < 0.01 vs. *nt* siRNA alone. ^#^*p* < 0.01 vs. rVvhA+ *nt* siRNA

Many bacterial stimuli regulate the PKC and MAPK pathways, which are interesting candidates as downstream mediators of ROS^8,25,26^. The rVvhA treatment significantly induced pan-PKC phosphorylation between 30 and 60 min (Fig. 3a), and that increase was blocked by melatonin treatment (Fig. 3b). In addition, we checked the effect of melatonin on VvhA-phosphorylated c-Src and NF-κB. However, melatonin failed to regulate c-Src phosphorylation induced by rVvhA, suggesting that melatonin uniquely regulates NF-κB-mediated cell death triggered by rVvhA (Sup. Fig. 5). Importantly, the inhibitory effect of melatonin was silenced by treatment with the siRNAs for *MT*_*2*_ and *Gαq* (Fig. 3c). The rVvhA treatment also increased the phosphorylation of JNK between 30 and 120 min, but it did not affect the phosphorylation of ERK or p38 MAPK (Fig. 3d); moreover, its effect at 30 min could be inhibited by melatonin treatment as well as by the silencing of PKCα, which is a major PKC isoform responsible for the rVvhA signaling pathway (Fig. 3e). The silencing of *MT*_*2*_ and *Gαq* by siRNAs had a significant inhibitory effect on the melatonin signaling pathway, blocking the JNK activation induced by rVvhA (Fig. 3f). The rVvhA increased the expressions of Bax, cleaved caspase-9, and cleaved caspase-3, but rVvhA-induced apoptotic signaling pathways were blocked by JNK inhibitor, SP600125 treatment (Fig. 3g). In addition, the inhibition of JNK signaling significantly abrogated the apoptotic effect of rVvhA (Fig. 3h). Taken together, the above results indicate that PKC-mediated JNK phosphorylation is required for the regulation of apoptotic cell death as evoked by rVvhA, and that the rVvhA signaling pathway can be negatively regulated by melatonin signaling pathways.

### Role of melatonin-inhibited JNK/c-Jun pathway in rVvhA-induced apoptosis

**Fig. 4:**
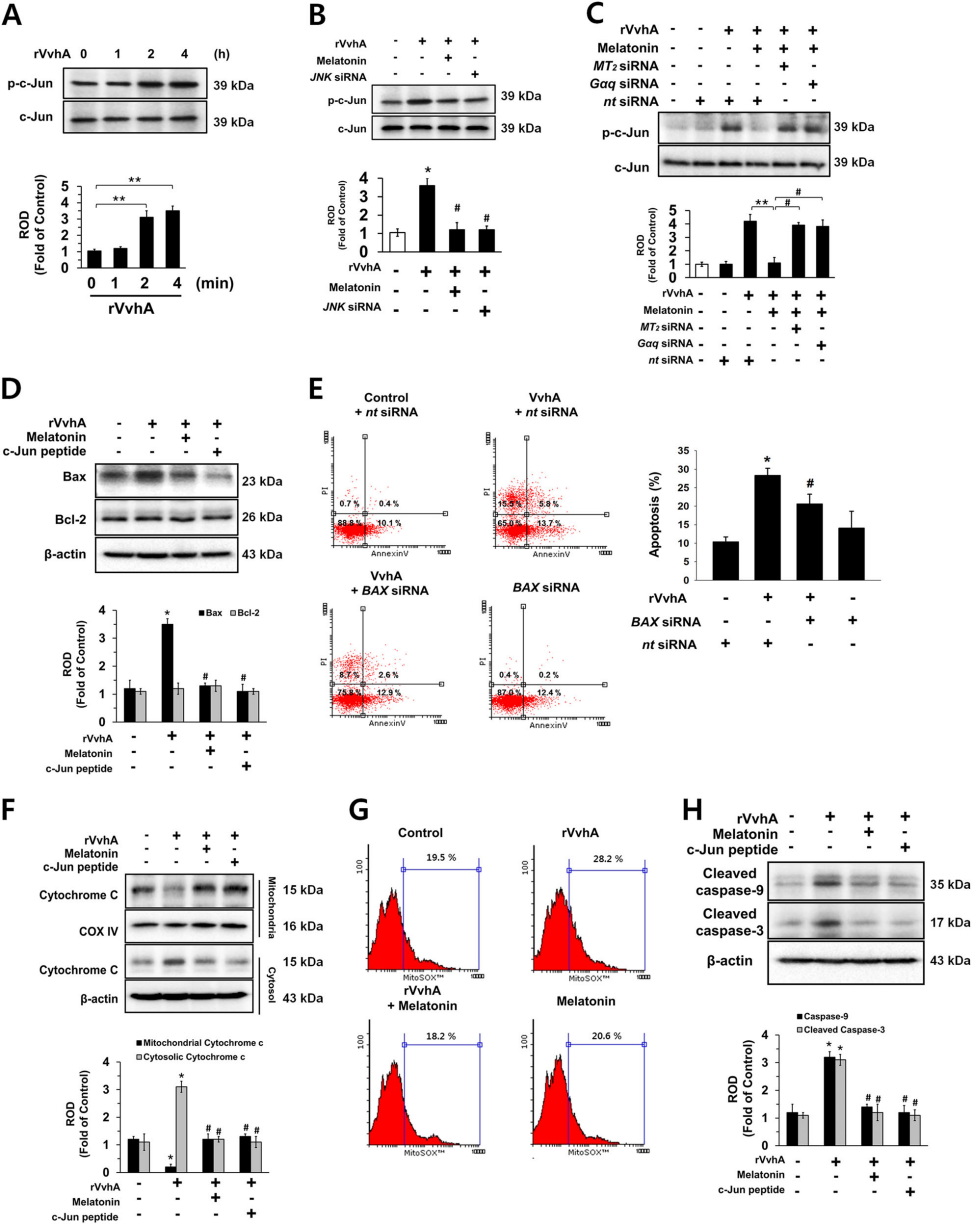
Regulatory effect of melatonin on apoptotic cell death mediated by c-Jun activation **a** Phosphorylation of c-Jun is shown. *n* = 3. **p < 0.01 vs. 0 min. **b** Phosphorylation of c-Jun in a cells treated with melatonin (1 µM) or transfected with siRNAs for JNK for 24 h prior to rVvhA exposure for 4 h is shown. *n* = 4. *p < 0.01 vs. control. ^#^*p* < 0.01 vs. rVvhA alone. **c** Cells transfected with siRNAs for MT_2_ or Gαq were incubated with melatonin (1 µM) for 30 min prior to rVvhA exposure for 4 h. The level of c-Jun phosphorylation is shown. Data represent the mean ± S.E. *n* = 4. **p < 0.01 vs. rVvhA+ *nt* siRNA. ^#^*p* < 0.01 vs. rVvhA+ melatonin+ *nt* siRNA. **d**, **e** Cells were incubated with c-Jun peptide (100 µM) and melatonin (1 µM) for 30 min prior to rVvhA exposure for 24 h. **d** Expressions of Bax and Bcl-2 are shown. *n* = 3. *p < 0.01 vs. control. ^#^*p* < 0.05 vs. rVvhA alone. **e** Cells transfected with *nt* or *BAX* siRNAs were incubated with melatonin (1 µM) for 30 min prior to rVvhA exposure for 24 h. Quantitative analysis of the percentage of apoptotic cells by flow cytometer analysis is shown. *n = *5. *p < 0.05 vs. *nt* siRNA. ^#^*p* < 0.05 vs. rVvhA+ *nt* siRNA. **f** Expressions of cytochrome C in the mitochondrial and cytosol fraction are shown. *n* = 3. *p < 0.01 vs. control. ^#^*p* < 0.01 vs. rVvhA alone. **g** Mitochondrial ROS production was measured by flow cytometer following staining with MitoSOX. The percentages of MitoSOX-positive cells analyzed by flowing software. *n* = 4. **h** Cells were incubated with c-Jun peptide (100 µM) and melatonin (1 µM) for 30 min prior to rVvhA exposure for 24 h. Expressions of caspase-9 and cleaved caspase-3 are shown. *n* = 3. *p < 0.05 vs. control. ^#^*p* < 0.05 vs. rVvhA alone.

The rVvhA treatment phosphorylated c-Jun, a downstream factor of JNK, phosphorylation between 2 and 4 h (Fig. 4a). In addition, treatment with melatonin as well as *JNK* siRNA significantly blocked rVvhA-induced c-Jun activation (Fig. 4b). The inhibitory effect of melatonin on phosphorylation of c-Jun was normalized by knockdown of MT_2_ and Gαq expression (Fig. 4c). In addition, the rVvhA exposure increased Bax expression but did not change Bcl-2 expression, suggesting that rVvhA alters the balance of Bcl-2/Bax (Fig. 4d). In addition, the rVvhA-induced increase in Bax was reversed by treatment with melatonin and c-Jun peptide. And, we investigated the effect of VvhA on conformational change of Bax in mitochondria as described earlier^27^. As shown in the Sup. Fig. 6, VvhA treatment triggered 6A7 epitope exposure of Bax in the mitochondria. And, we further confirmed that *BAX* siRNA transfection significantly suppressed rVvhA-induced ROS production (Supplemental Fig. 7) and apoptosis (Fig. 4e). The rVvhA induced cytochrome c release from mitochondria to cytosol, and that release was inhibited by treatment with melatonin and c-Jun peptide (Fig. 4f). Furthermore, we investigated mitochondrial ROS production in VvhA or melatonin treated cells by using MitoSOX, a mitochondrial ROS-specific fluorescent dye, to confirm the effect of melatonin on rVvhA-induced mitochondrial apoptosis. The result revealed that rVvhA increases mitochondrial ROS production, which was negatively regulated by melatonin treatment (Fig. 4g). Consistent with these results, rVvhA stimulated the expression of cleaved caspase-9 and cleaved caspase-3, which were blocked by treatment with melatonin and c-Jun peptide (Fig. 4h). Furthermore, we observed that VvhA is not presented in the mitochondrial fraction compared to non-mitochondrial fraction (Supplemental Fig. 8). Based upon those results, we newly present that melatonin pretreatment or inhibition of JNK/c-Jun/Bax pathway has a protective role in rVvhA-induced mitochondrial ROS production, which may be associated with rVvhA-induced mitochondrial apoptosis.

### Regulatory effect of melatonin on autophagic cell death mediated by Bcl-2 phosphorylation

**Fig. 5:**
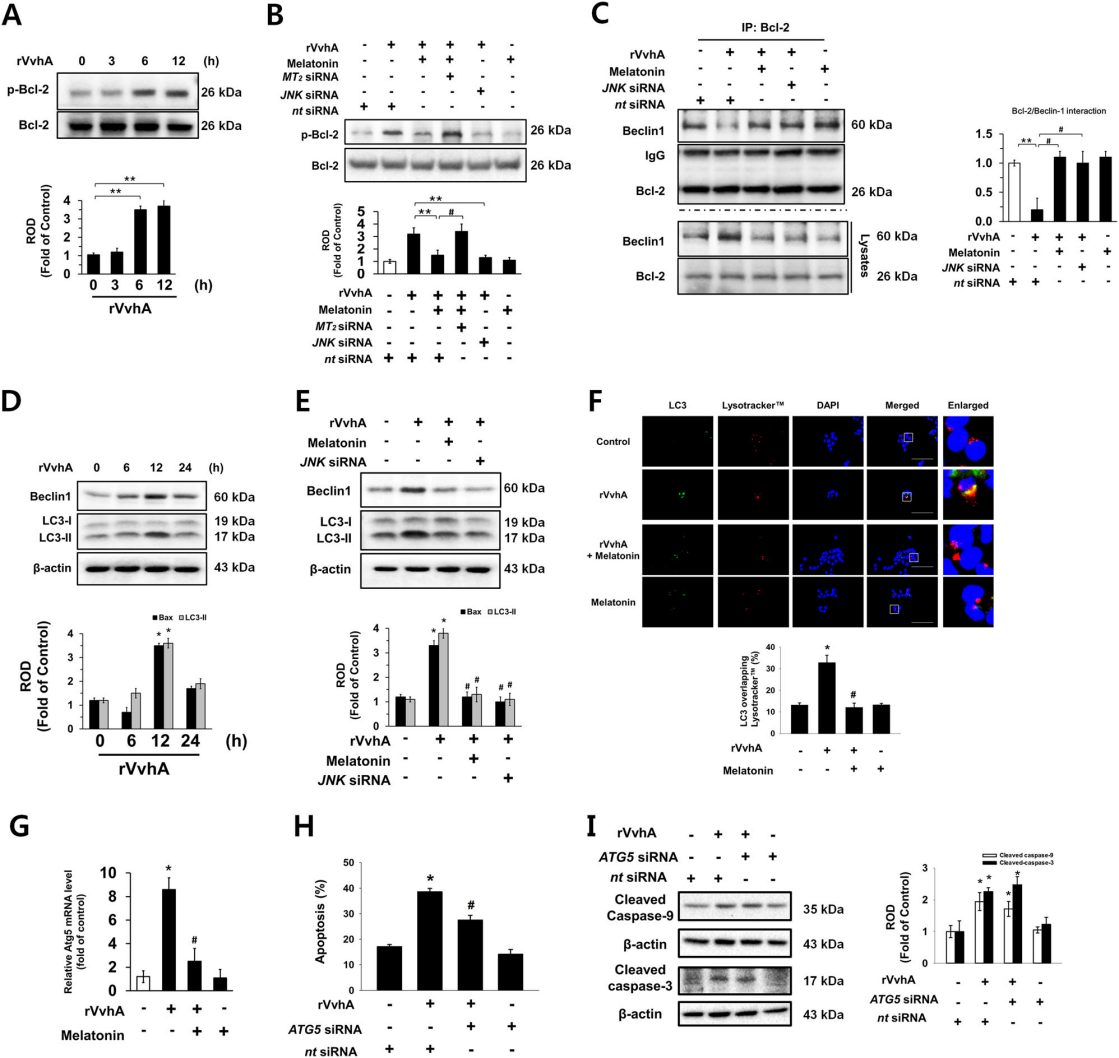
Regulatory effect of melatonin on autophagic apoptosis mediated by Bcl-2 phosphorylation **a** Phosphorylation of Bcl-2 is shown. *n* = 3. **p < 0.01 vs. 0 h. **b** Cells transfected with siRNAs for MT_2_ and JNK were incubated with melatonin (1 µM) for 30 min prior to rVvhA exposure for 12 h. The level of Bcl-2 phosphorylation is shown. Data represent the mean ± S.E. *n* = 4. **p < 0.01 vs. rVvhA+ *nt* siRNA. ^#^*p* < 0.05 vs. rVvhA+ melatonin+ *nt* siRNA. **c** Bcl-2 co-immunoprecipitated with Beclin1 (upper panel). The level of Beclin1 and Bcl-2 in total cell lysates is shown in the bottom panel. *n = *3. **p < 0.05 vs. *nt* siRNA. ^#^*p* < 0.05 vs. rVvhA+ *nt* siRNA. **d** Expressions of Beclin-1 and LC-3 are shown. *n* = 3. *p < 0.01 vs. 0 h. **e** Cells transfected with siRNA for JNK were incubated with melatonin (1 µM) for 30 min prior to rVvhA exposure for 12 h. Expressions of Beclin-1 and LC-3 are shown. *n* = 4. *p < 0.05 vs. control. ^#^*p* < 0.05 vs. rVvhA. **f** The cells were immunostained with LC-3 antibody (green), Lysotracker (red) and DAPI (blue). The percentage of co-localization of LC-3 with Lysotracker was measured by Metamorph software. *n* = 4. Magnification is 400× objective, scale bars are 5 μm. Data are presented as a mean ± S.E. *n* = 4. *p < 0.05 vs. control. ^#^*p* < 0.05 vs. rVvhA alone. **g** The expression of *ATG5* mRNA was evaluated by qRT-PCR in the cells treated with melatonin (1 µM) for 30 min prior to rVvhA exposure for 24 h. Data present as a mean ± S.E. *n* = 4. *p < 0.01 vs. control. ^#^*p* < 0.01 vs. rVvhA alone. **h, i** Cells transfected with siRNA for Atg5 were incubated with melatonin (1 µM) for 30 min prior to rVvhA exposure for 24 h. Quantitative analysis of the percentage of apoptotic cells by flow cytometer analysis is shown. *n = *4. *p < 0.05 vs. *nt* siRNA. ^#^*p* < 0.05 vs. rVvhA+ *nt* siRNA. The level of cleaved caspase-9 and -3 are shown. Data are presented as a mean ± S.E. *p < 0.05 vs. *nt* siRNA. ^#^*p* < 0.05 vs. rVvhA+ *nt* siRNA

Given that there was no change in the expression of Bcl-2 following rVvhA treatment, it is possible that rVvhA regulates Bcl-2 phosphorylation, which is responsible for the occurrence of Beclin-1-mediated autophagy^28^. An increase in the phosphorylated form of Bcl-2 without a change in its expression level was observed after 6–12 h of incubation with 50 pg/mL of rVvhA (Fig. 5a). Melatonin significantly blocked the Bcl-2 activation induced by rVvhA via MT_2_ (Fig. 5b). Importantly, phosphorylation of Bcl-2 was significantly inhibited by knockdown of JNK (Fig. 5b). We further observed that the interaction between Bcl-2 and Beclin-1 was inhibited by rVvhA treatment (Fig. 5c). In contrast, the interactions induced by rVvhA were significantly recovered by treatment with melatonin or knockdown of JNK expression. Consistent with these results, rVvhA stimulated the expression of Beclin-1 and LC-3 (Fig. 5d), and those expression increases were blocked by treatment with melatonin and *JNK* siRNA (Fig. 5e), indicating that melatonin may have a unique signaling path in the regulation of autophagic cell death mediated by JNK activation in rVvhA-treated HCT116. In addition, we observed that rVvhA treatment reduced p62 expression, which was reversed by melatonin pretreatment (Supplemental Fig. 9), and melatonin inhibited rVvhA-induced LC-3 expression in INT407 cells (Supplemental Fig. 2B). These data indicate that melatonin blocks rVvhA-induced autophagy. Immunocytochemstry results also showed that rVvhA treatment increased the co-localization of LC-3 puncta, autophagosome marker, with Lysotracker-positive puncta in cells, which was revered by melatonin pretreatment (Fig. 5f). Interestingly, melatonin significantly blocked the expression of *ATG5* induced by rVvhA (Fig. 5g), and that knockdown of *ATG5* also inhibited apoptotic cell death triggered by rVvhA (Fig. 5h). In addition, *ATG5* siRNA transfection did not affect the rVvhA-induced caspase-9 and caspase-3 cleavages (Fig. 5i). These results indicate that melatonin inhibits autophagic cell death induced by rVvhA by regulating Bcl-2 phosphorylation and Bcl-2-Beclin-1 interaction in HCT116 cells.

## Discussion

Our results demonstrate that a melatonin pretreatment can prevent cell death induced by *V. vulnificus* VvhA, and that MT_2_ coupling with NCF-1 appears to have a major role in the prevention of apoptotic and autophagic cell death mediated by ROS production and JNK activation in colonic epithelial cells. The physiological meaning of melatonin in *V.vulnificus* infection can be explained as a host defense trigger. Indeed, previous reports reported the melatonin act as a signal molecule for ROS or nitric oxide-induced host defense response against bacterial infection^29,30^. In addition, another study showed the protective effect of melatonin to resist bacterial infection and presented the melatonin as a useful therapeutic drug for sepsis and septic injury^31^. Given that MT_2_ expression is limited in HCT116 cells, our results indicate that MT_2_ is the major melatonin receptor that regulates the anti-apoptotic signaling pathway. Moreover, we previously showed that *V. vulnificus* effectively generates mitochondria-mediated cell death by producing relevant cytolysin VvhA with modes of action that differ from those of EPEC and *Helicobacter pylori*^8^. Thus, pre-activation of HCT116 cells by melatonin may offer a means of improving the protective potency of host cells against *V. vulnificus* cytolysin. Concerning the concentration of melatonin, it was previously reported that the physiological levels of melatonin are in the picomolar and low nanomolar range in blood, whereas the melatonin concentrations in some tissues and cells can be exceptionally high^32^. Thus, the differences in concentrations of melatonin and application times in this and other experiments justify, in part, the discrepancies among the results of several studies^33–35^. We believe that these broad effects of melatonin are in part due to the presence of multiple melatonin receptors in different types of cells, thus outcomes may vary depending on the cellular concept.

A key observation in our study is that melatonin induces NCF-1 binding to MT_2_ in non-lipid rafts to block ROS production in HCT116 cells treated with rVvhA. Given that caveolin-1 is constitutively expressed in lipid rafts to maintain cell homeostasis forming caveolin-1-mediated caveolae^36^, it is possible that individual lipid rafts with caveolin-1 attached are present in fraction 4. These individual lipid rafts have been described as dynamic microdomains and carry several membrane-bound or attached proteins or enzymes such as G-proteins, protein kinases, and NOX enzymes^36^. Interestingly, an increase in the level of NCF-1 and Rac1 together with caveolin-1, appeared in fraction 3 after rVvhA treatment, suggesting that rVvhA enhances the recruitment of NOX enzymes into a caveolin-1-enriched component to initiate the production of ROS. However, our results revealed that melatonin induced the movement and clustering of MT_2_ and NCF-1 into non-lipid raft parts to block the aggregation of lipid raft components responsible for ROS production induced by rVvhA and restored the membrane locations of caveolin-1 and Rac1. Thus, indicating that melatonin is a functional agent preventing the virulence effect of rVvhA by regulating the organization of rafts and the spatial distribution of MT_2_ and NCF-1. The MT_2_ receptor initially couples with heterotrimeric Gα proteins. It was shown previously that MT_2_ couples with mainly Gαi and Gq, whereas MT_1_ interacts with Gαi, Gαq, and Gαs^24,37,38^. In HCT116 cells, however, we found that ROS production and apoptotic cell death mediated by MT_2_ were selectively regulated by Gαq, not by Gαi or Gα12. These results are supported by those in a previous study in which Gαq subunit-linked signal transduction was found to be a critical step in the regulation of melatonin-mediated pancreatic glucagon secretion and stem cell migration^24,39^.

The ROS generated by a bacterial infection have been shown to induce oxidative inactivation of several proteins harboring oxidant-sensitive thiol groups and of the ubiquitin-proteasome pathway^40^, thereby activating many redox-sensitive proteins, including regulators of MAP kinase (MAPK) pathways and several components involved in c-Jun activation^41^. Given the critical role of PKC in promoting the cell death process induced by ROS during EPEC, *Clostridium perfringens*, and *V. vulnificus* infections^8,25,42^, our current results indicate that melatonin acting through MT_2_ and Gαq may inhibit ROS production responsible for PKC activation to block the rVvhA-induced apoptotic cell death process. Although it has been known that G protein coupled receptor-Gαq signaling activates PKC via inositol triphosphate and diacylglycerol, the effect of melatonin on PKC activation appears cell type specific^24,43,44^. Therefore, we propose that MT_2_/Gαq/PKC signaling is overwhelmed by suppression of ROS production by melatonin’s action in the NCF-1. Despite the frequent involvement of ERK and p38 MAPK in the ROS signaling pathway induced by *H. pylori* infection^45^, an earlier report showed that the JNK pathway induced by ROS can influence c-Jun activation in promoting apoptotic cell death^46^. Our current results reveal that rVvhA uniquely regulates apoptotic cell death through activation of a JNK-mediated c-Jun pathway, and such activation was inhibited by melatonin treatment. However, our data also showed JNK inhibitor does not completely inhibit the rVvhA-mediated apoptosis. It is possible that VvhA also induces JNK-independent apoptotic pathway for perturbations in ER function (ER stress), which is critical for cell survival^47^. This is further supported by previous results showing that rVvhA significantly induces CCAAT/enhancer binding protein (C/EBP) homologous protein (CHOP), which is one of the components of the ER stress-mediated cell death pathway^13^.

Having determined that *H. pylori* induces oxidative stress leading to apoptotic cell death via Bax oligomerization, Bax has been suggested as a primary determining factor of the degree of susceptibility to apoptosis^48–51^. In addition, a previous study showed that JNK-increased c-Jun activity induces transcription of the *BAX* gene in response to butyric acid^52^. Although there has been no report showing relationship between VvhA-induced Bax and mitochondrial ROS production, a previous report demonstrating the Bax-induced mitochondrial dysfunction and apoptosis by bacterial infection suggests a possibility of the important role of VvhA-induced Bax in mitochondrial ROS production^53^. This study shows that melatonin blocks Bax expression and the release of mitochondrial cytochrome c, which are unique downstream events in the rVvhA-evoked mitochondrial apoptotic pathway accompanying the production of mitochondrial ROS as well as the cleavage of caspase-9 and caspase-3. Interestingly, a pore-forming α-toxin from *Staphylococcus aureus* has been shown to induce massive necrosis in the absence of an apoptotic process^54^, whereas EPEC was shown to disrupt the mitochondrial membrane potential, resulting in the release of cytochrome c and apoptosis^55^. Thus, these results imply that melatonin has a unique function to block infectious mechanisms caused by the pore-forming toxin rVvhA to prevent activation of the mitochondrial apoptotic pathway in HCT116 cells.

In addition to results showing JNK mediation of the mitochondrial apoptotic pathway, previous and present results show that JNK participates in multiple stimulation-induced autophagic events and modulates autophagy at multiple regulatory levels^28,56^. Specifically, JNK stimulates the dissociation of Beclin1 from Bcl-2 after the phosphorylation of amino acid residues on Bcl-2 thereby inducing autophagy^28,56^. The present study identified that rVvhA failed to regulate Bcl-2 expression; instead, it stimulated Bcl-2 phosphorylation mediated by JNK and triggered the dissociation of Beclin1 from Bcl-2 in the promotion of autophagy-mediated cell death. Importantly, melatonin pretreatment resulted in inhibition of Bcl-2 phosphorylation and Bcl-2-Beclin1 dissociation thereby preventing autophagic cell death. Autophagic cell death is regulated by rVvhA in macrophages via Atg5^13^, which is critical for the formation of autophagosomes, and it was previously reported that JNK activation is necessary for up-regulation of *ATG5* and *BECLIN1* as well as autophagy-mediated cell death^57^. Thus, our results showing an inhibitory effect of melatonin on autophagic cell death indicates that melatonin prevents the rVvhA signaling pathway, which stimulates the JNK activation responsible for cell death mediated by Bcl-2 phosphorylation-mediated autophagic pathway and the c-Jun-mediated mitochondrial apoptotic pathway in HCT116 cells. Although our study shows that both mitochondrial apoptosis and autophagic cell death pathways are critical for rVvhA-induced intestinal cell apoptosis, further investigation will be needed to determine the relative contribution of each pathway to rVvhA-induced cell death. In the present study, we already have shown that rVvhA-induced apoptotic pathway including, mitochondrial damage and caspase activation is inhibited by treatment with *JNK* siRNA as well as JNK inhibitor (Figs. 3–5). In addition, the inhibitory effect of melatonin on the JNK activation is important for suppression of rVvhA-induced apoptotic pathway. These mean that JNK inactivation or silencing reduced rVvhA-triggered mitochondrial apoptotic cell death, suggesting that JNK as key regulator and potential target for VvhA-induced apoptosis.

**Fig. 6:**
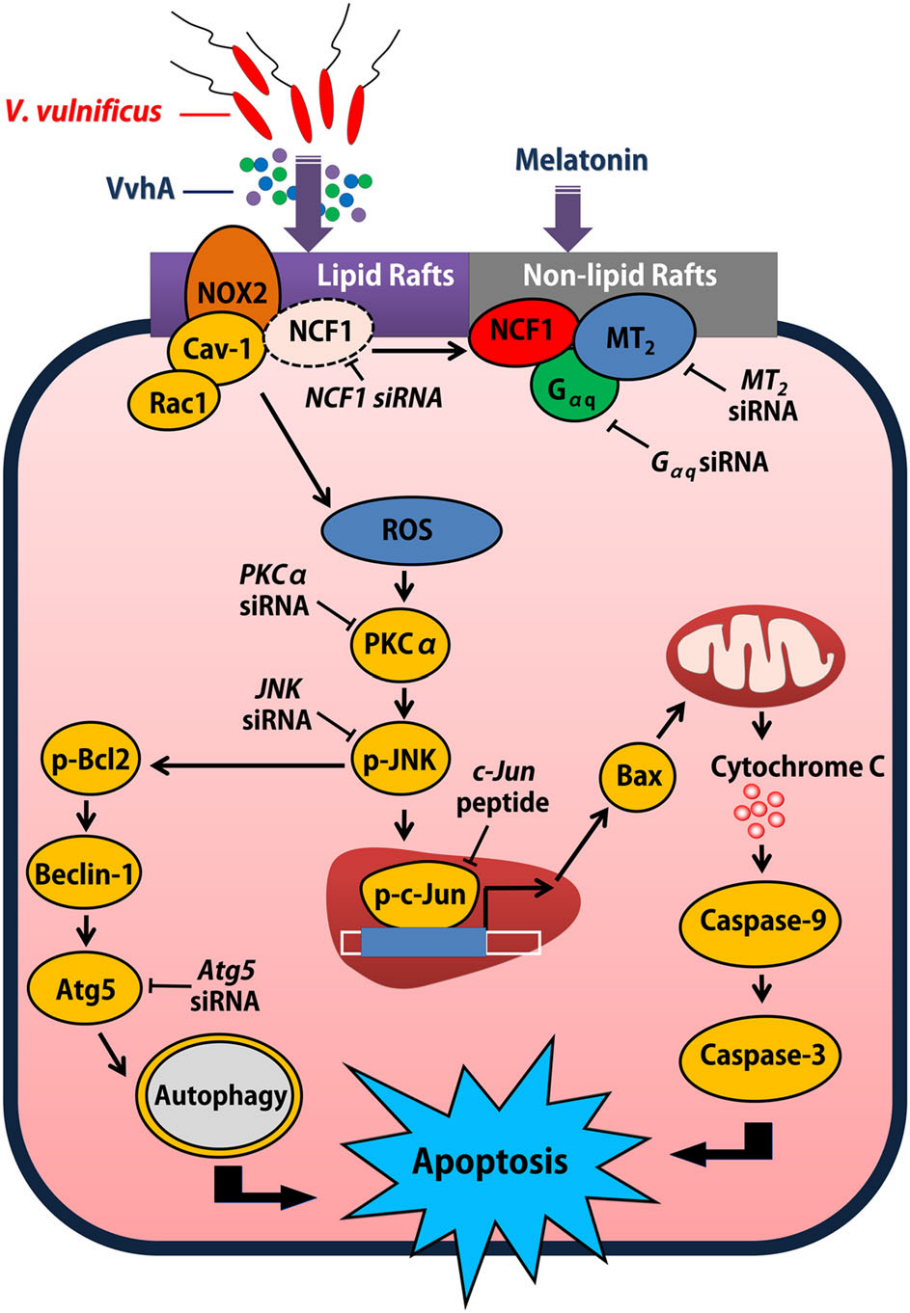
A hypothetical model for melatonin-induced signaling pathway against V. vulnificus infection Recruitment of NCF-1 by melatonin into the MT_2_ receptor blocks *V. vulnificus-*induced NOX2-dependent ROS production, which inhibits PKCα/JNK signaling pathway-activated mitochondrial apoptosis and autophagic cell death

To our knowledge, this is the first report demonstrating melatonin’s role in the VvhA-induced formation of the NCF-1/NOX2 complex and suggesting a novel action of melatonin in the control of ROS production induced by VvhA. In conclusion, melatonin signaling via MT_2_ stimulates NCF-1 recruitment into non-lipid rafts from lipid rafts to block the ROS-mediated JNK pathway, which leads to prevention of rVvhA-induced apoptosis and autophagic intestinal cell death (Fig. 6). Moreover, our results provide important insight into the potential for development of therapeutic strategies and agents for *V. vulnificus* infection treatment.

## Electronic supplementary material

Supplemental information
