# Supplementary material for: Melatonin inhibits apoptotic cell death induced by Vibrio vulnificus VvhA via melatonin receptor 2 coupling with NCF-1
Source: Cell Death Dis. 2018 Jan 19;9(2):48. doi: 10.1038/s41419-017-0083-7 (PMC5833450; doi:10.1038/s41419-017-0083-7)
Supplement: Supplementary file 1 — Supplemental information [file 41419_2017_83_MOESM1_ESM.docx]

**Melatonin inhibits apoptotic cell death induced by *Vibrio vulnificus* VvhA via melatonin receptor 2 coupling with NCF-1**

**Supplemental Figures**


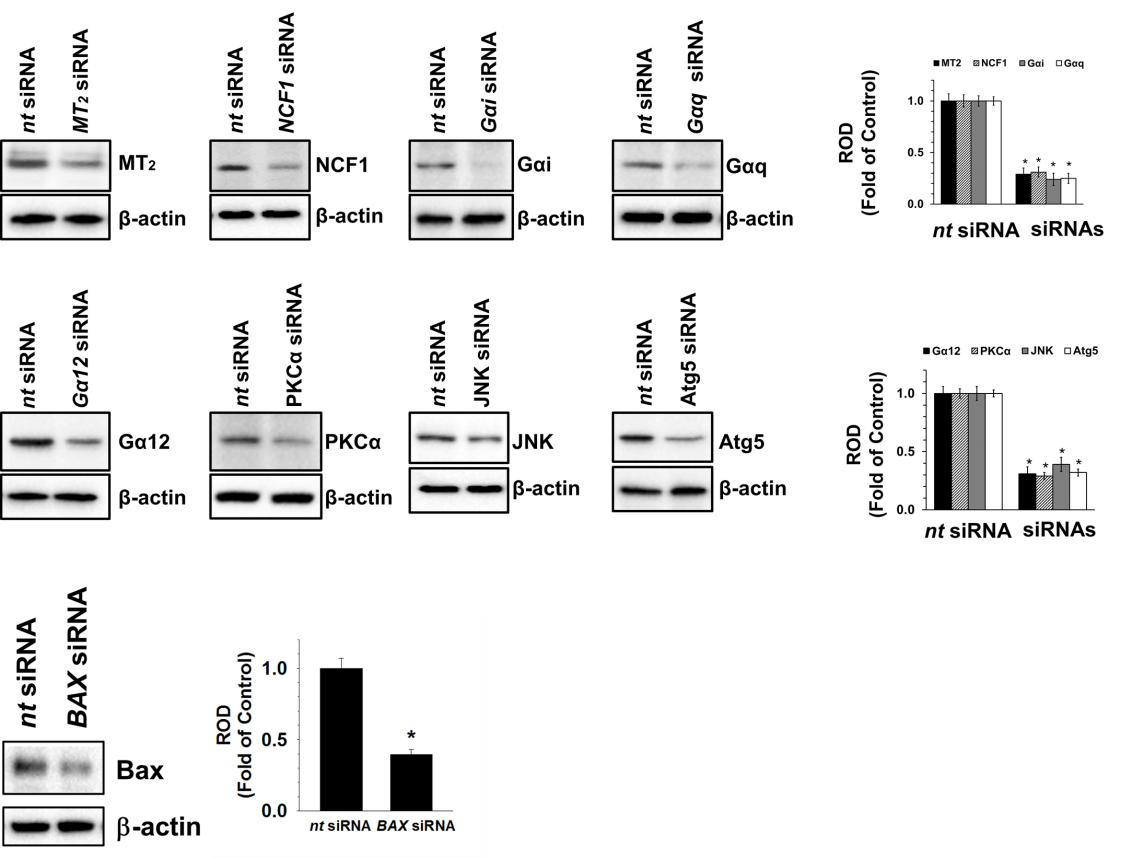


**Supplemental figure 1. Effect of siRNA on target proteins.** Cells were transfected with *MT_2_, NCF1, Gαi, Gαq, Gα12, PKCα, JNK, ATG5, BAX* or *non-targeting* (*nt*) siRNA using HiPerFect Transfection Reagent. Protein expressions were analyzed by using Western blot. The siRNA efficacies for MT_2_, NCF1, Gαi, Gαq, Gα12, PKCα, JNK, Atg5, and Bax determined by Western blot were 71, 69, 76, 75, 69, 71, 61, 68, and 60%, respectively. Error bars represent the means ± S.E. from three independent experiments. *, *P <* 0.05 vs *nt* siRNA.


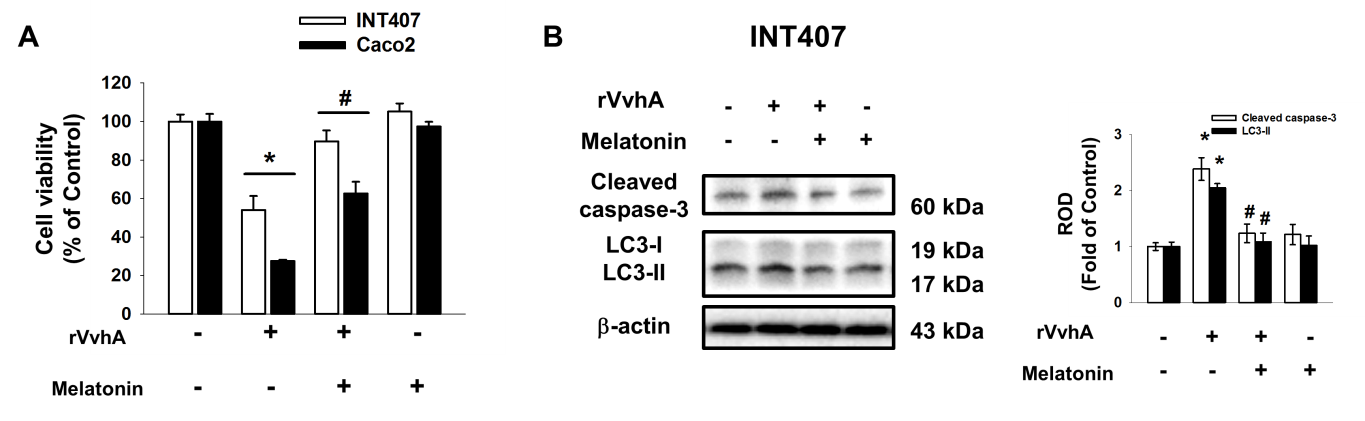


**Supplemental figure 2. Effect of melatonin on VvhA-induced apoptosis and autophagy in INT407 and Caco2 cell lines**. (A) INT407 and Caco2 cells were pretreated with melatonin (1 μM) for 30 min prior to rVvhA (50 pg/mL) exposure for 24 h. Cell viability was measured by using EZ-CYTOX™ cell viability assay. *n*=8. *, *p* < 0.05 vs. control. #, *p* < 0.05 vs. rVvhA alone. (B) INT407 cells were pretreated with melatonin (1 μM) for 30 min prior to rVvhA (50 pg/mL) exposure for 6h. Expressions of cleaved caspase-3, LC3 and β-actin are shown. Data are presented as a mean ± S.E. *n*=3. *, *p* < 0.05 vs. control. #, *p* < 0.05 vs. rVvhA alone.


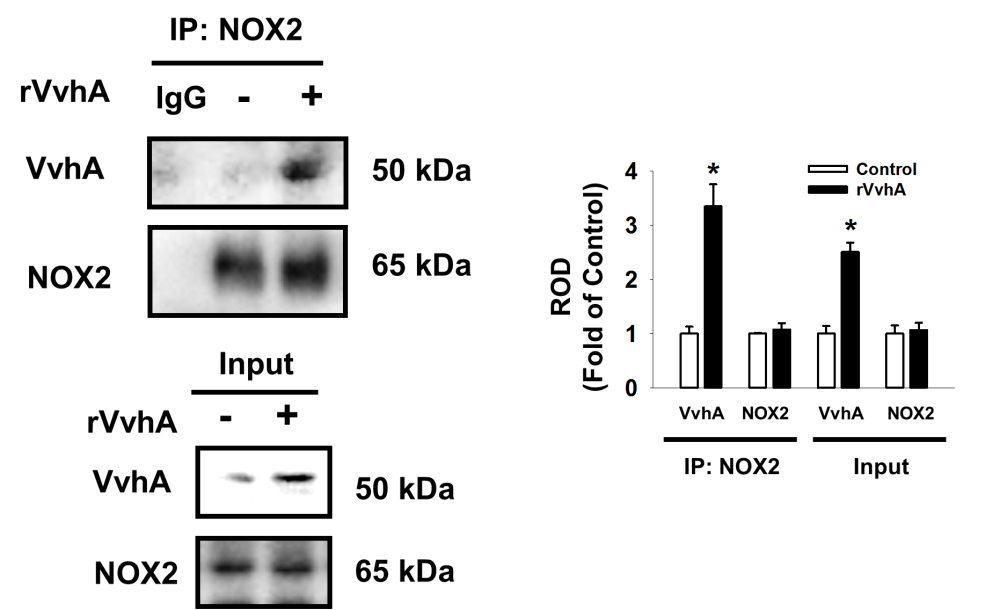


**Supplemental figure 3. Interaction of VvhA with NOX2.** Cells were incubated with rVvhA (50 pg/mL) for 3h. Cells were immunoprecipitated with NOX2-specific antibody. Expressions of NOX2, VvhA and β-actin are shown. *n*=3. Data are presented as a mean ± S.E. *, *p* < 0.05 vs. control.


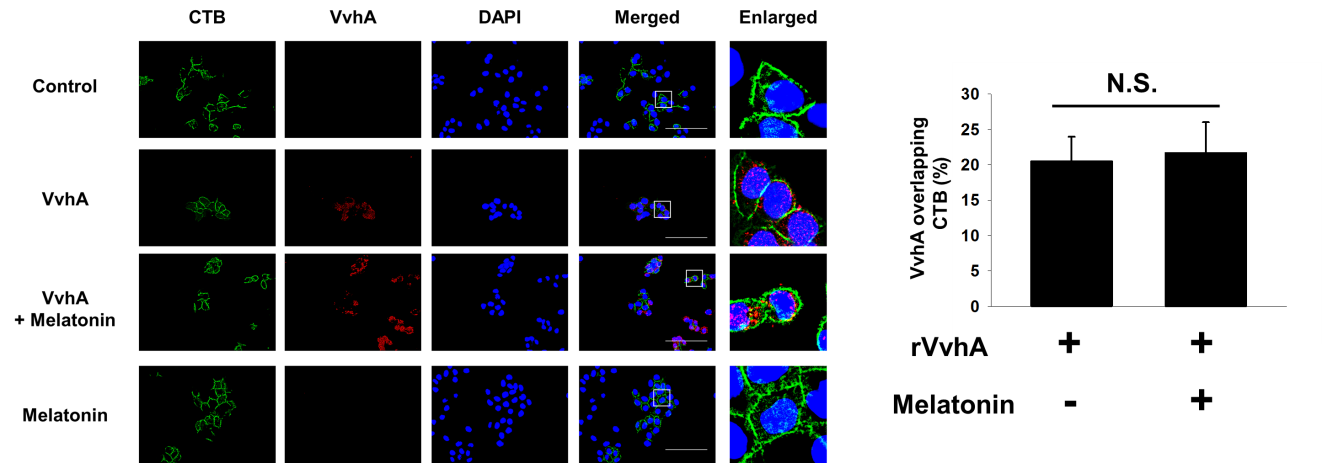


**Supplemental figure 4. Effect of melatonin on VvhA binding to lipid raft.** Cells were pretreated with melatonin (1 μM) for 30 min prior to VvhA (50 pg/mL) exposure at 4°C for 30 min. Cells were immunostained with CTB (green), VvhA antibody (red) and DAPI (blue). Immunofluorescence images are representative. *n*=4. Magnification is 400 × objective, scale bars are 5 μm. Data are presented as a mean ± S.E. *, *p* < 0.05 vs. control. N.S. is not significant.


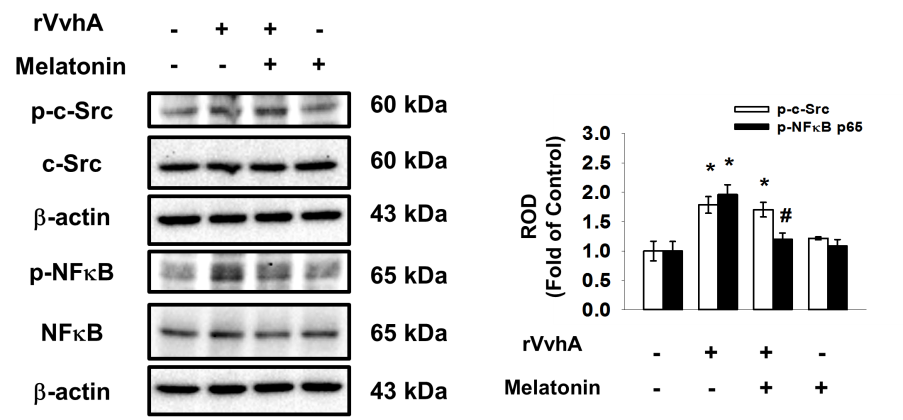


**Supplemental** f**igure 5. Effect of melatonin on rVvhA-induced c-Src and NF-κB phosphorylations.** Cells were pretreated with melatonin (1 μM) for 30 min prior to VvhA (50 pg/mL) exposure for 30 min. Expressions of p-c-Src (Tyr418), c-Src, p-NF-κB (Ser536), NF-κB and β-actin are shown. Data are presented as a mean ± S.E. *n*=4. *, *p* < 0.05 vs. control. #, *p* < 0.05 vs. rVvhA alone.


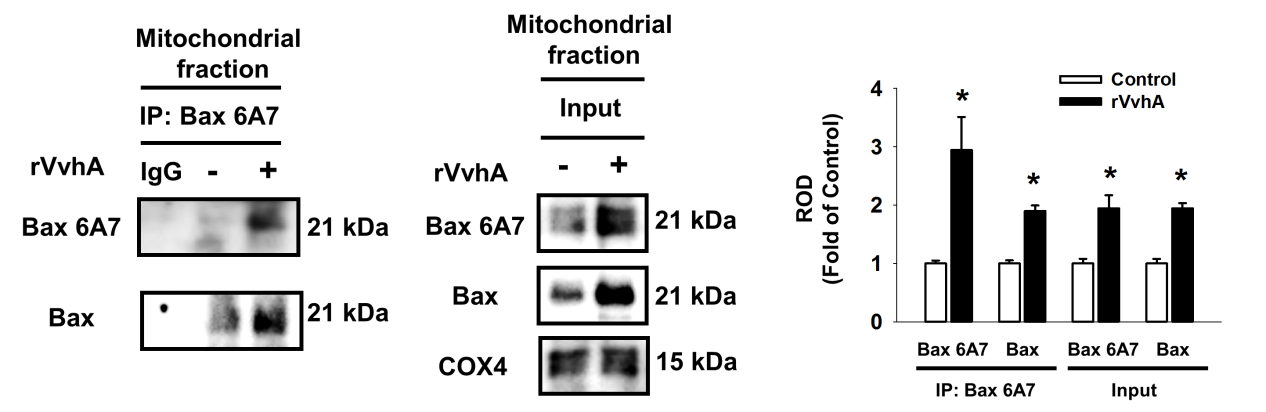


**Supplemental figure 6. Effect of VvhA on Bax conformational change in mitochondria.** Cells were exposed to VvhA (50 pg/mL) for 24 h. Mitochondrial fractionized samples were immunoprecipitated with Bax 6A7 antibody. Expressions of conformational change form of Bax (6A7) and total Bax are shown. Data are presented the mean ± S.E. *n*=4. *, *p* < 0.05 vs. control.


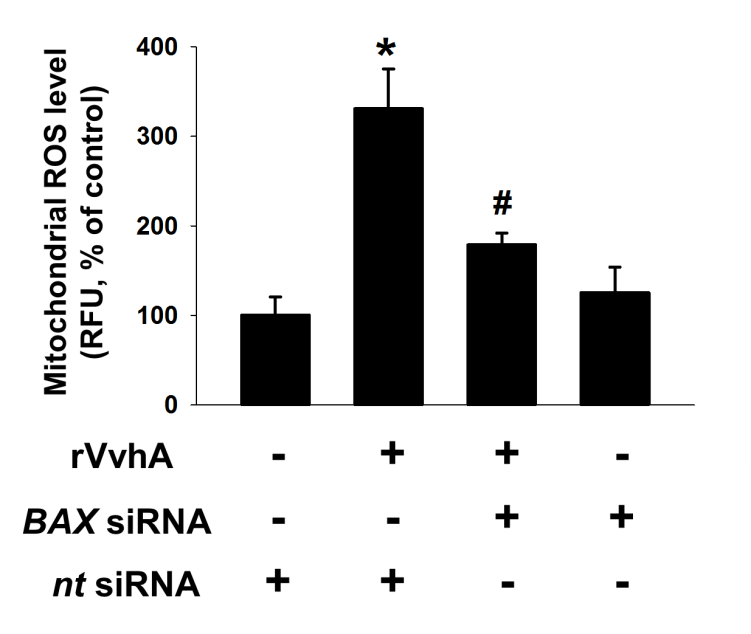


**Supplemental figure 7. Role of Bax in VvhA-induced mitochondrial ROS production.** Cells transfected with *BAX* or *nt* siRNAs were exposed to rVvhA (50 pg/mL) for 12 h. The level of ROS production is shown. Data are presented as a mean ± S.E. *n*=8. *, *p* < 0.05 vs. *nt* siRNA alone. *^#^*, *p* < 0.05 vs. rVvhA + *nt* siRNA.


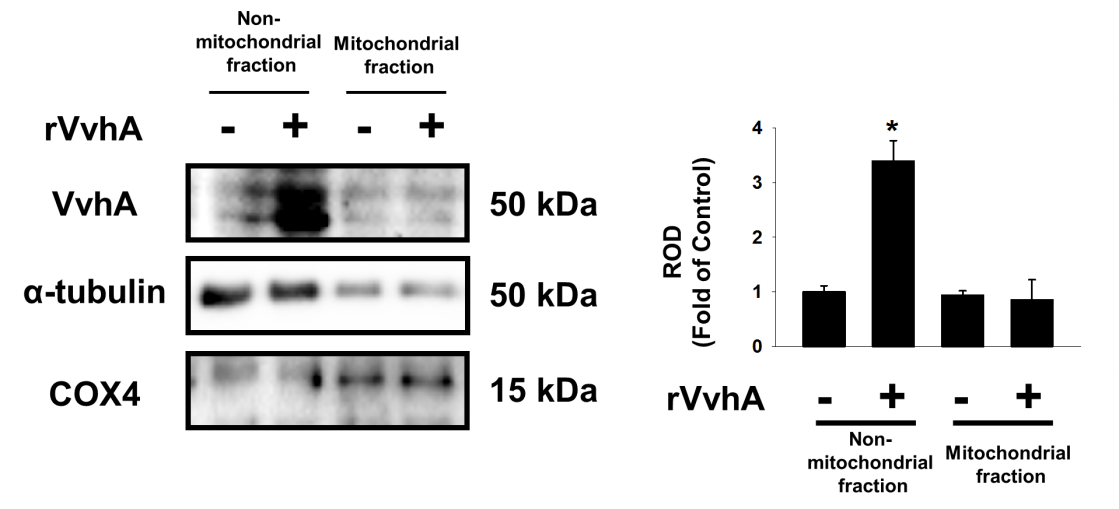


**Supplemental figure 8. Translocation of VvhA into the mitochondria.** Cells were treated with rVvhA (50 pg/mL) for 24 h. Expressions of VvhA, α-tubulin and COX4 in the non-mitochondrial and mitochondrial samples are shown. Data are presented as a mean ± S.E. *n*=4 *, *p* < 0.05 vs. control of non-mitochondrial fraction.


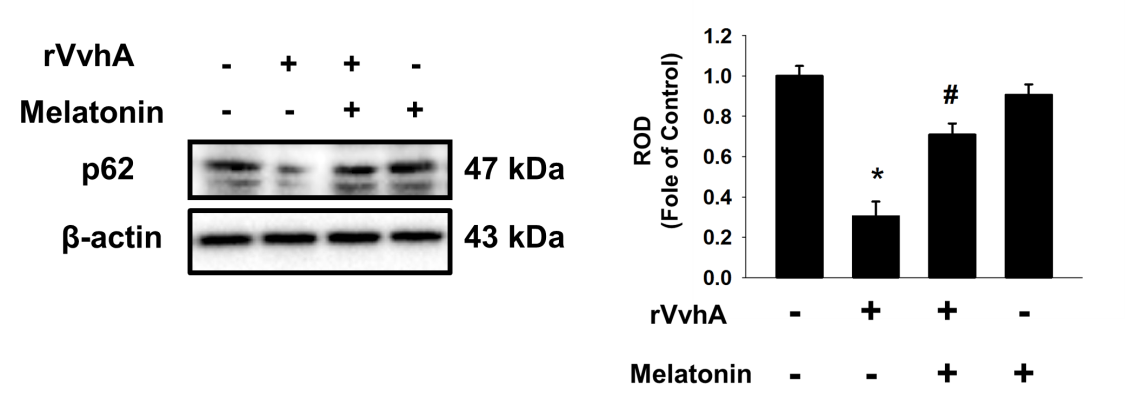


**Supplemental figure 9.** Effect of melatoin on rVvhA-induced p62 expression. Cells were pretreated with melatonin (1 μM) for 30 min prior to rVvhA (50 pg/mL) exposure for 6h. Expression of p62 and β-actin are shown. Data are presented as a mean ± S.E. *n*=4. *, p < 0.05 vs. control. #, p < 0.05 vs. rVvhA alone.

**Supplemental Tables**

**Supplemental Table 1. Oligonucleotides used in this study**

| **Name** | **Oligonucleotide Sequence (5' → 3')^a, b^** | **Use** |
| --- | --- | --- |
| **For GbpA overexpression** | | |
| Vvh-F | GGGCATATGCTTAATAACAAAAATAGAAATGTAG | Amplification of *VvhBA* ORF |
| Vvh-R | AAAACTCGAGTTTGACTTGTTGTAATGTGG |  |

^a^ The oligonucleotides were designed using the *V. vulnificus* MO6-24/O genomic sequence (GenBank^TM^ accession number CP002469 and CP002470, www.ncbi.nlm.nih.gov).

^b^ Regions of oligonucleotides not complementary to the corresponding genes are underlined

**Supplemental Table 2. Plasmids and bacterial strains used in this study**

| **Strain or plasmid** | **Relevant characteristics ^a^** | **Reference or source** |
| --- | --- | --- |
| **Bacterial strains** | |  |
| *E. coli* |  |  |
| BL21 (DE3) | F^-^ *ompT* *hsdS_B_* (r_B_^-^m_B_^-^) *gal dcm* (DE3) | Laboratory collection |
| **Plasmids** |  |  |
| pET29a(+) | His_6_ tag fusion expression vector; Km^r^ | Novagen |
| pKS1201 | pET29a(+) with VvhBA; Km^r^ | This study |

^a^ Km^r^, kanamycin resistan

**Supplementary Table 3. Sequences of primers used for RT-PCR**

| Gene name | Species | Identification | Sequence (5'-3') | Size (bp) |
| --- | --- | --- | --- | --- |
| *MTNR1A* | Human | Sense | TGTCGATATTTAACAACGGGTGG | 108 |
|  |  | Antisense | CGATGCCGGTGATGTTGAA |  |
| *MTNR1B* | Human | Sense | GCATGGCCTACCACCGAATC | 201 |
|  |  | Antisense | AATAGATGCGTGGGTCGTACT |  |
| *ATG5* | Human | Sense | TGGGATTGCAAAATGACAGA | 236 |
|  |  | Antisense | TTCCCCATCTTCAGGATCAA |  |
| *ACTB* | Human | Sense | AACCGCGAGAAGATGACC | 351 |
|  |  | Antisense | AGCAGCCGTGGCCATCTC |  |
